# The Ego Phenomenon and the Doping Problem in Sport: A Historical Conceptual Analysis

**DOI:** 10.3389/fspor.2021.728506

**Published:** 2021-11-10

**Authors:** Barend J. M. Steyn, Kim Nolte

**Affiliations:** ^1^Department of Psychology, University of Pretoria, Pretoria, South Africa; ^2^Department of Physiology, Biokinetics and Sport Science Division, University of Pretoria, Pretoria, South Africa

**Keywords:** ego phenomenon, outcome orientation, fixed mindset, social comparison, doping

## Abstract

The concept of ego has various meanings in the field of psychology, depending on the paradigmatic and theoretical framework point of departure. The ego phenomenon as operationalized and measured in the theoretical framework of goal orientation will be the contextual framework for a historical conceptual analysis. In the past three decades, research in the theoretical framework of goal orientation has revealed a positive relationship between ego involvement and the tendency to use the prohibited substances to enhance performance in sport. The concept of the ego phenomenon as operationalized within goal orientation theory and meanings attached to the concept can be connected to the historic oriental writings that were written ~2,500 years ago. These attached meanings to the ego phenomenon include elements of extreme competitiveness and outcome orientation, as well as social comparisons and the external norms for the measurement of success and failure. These meanings can be traced back to the classical works involving the Bhagavad Gita, the Tao Te Ching, and the Eastern Origins of Mindfulness that are part of the broader Buddhist philosophical system. Meister Eckhart, a 12th century German theologian, in his significant contribution on the analysis of the *having mode* as opposed to the *being mode* also provides insight into the ego phenomenon that can explain why the ego phenomenon can be linked to some of the deeper psychological motives of using the prohibited substances. The researchers in psychology do not yet have a full understanding of why certain athletes dope or have a susceptibility to use the prohibited substances or performance enhancing drugs (PEDs) and thus the motivation for this historical conceptual analysis of the ego phenomenon. Therefore, this article aimed to deepen the understanding of psychological motives of the athletes who exhibit tendencies toward cheating in general and the proclivity to use the prohibited substances.

## Introduction

Doping in sport is a phenomenon that can be traced back to the advent of sport. Evidence has been found that the Greek athletes experimented with ingesting certain plants; they believed that the plant substance could improve their performance during the Olympic Games in the third century BC (Murphy, 2005). The thought of enhancing performance (doping) has accompanied sport throughout history and has developed in a very complex phenomenon that affects all the sport codes internationally and on different levels (Prokop, 1970). The World Anti-Doping Agency (WADA) has developed a variety of strategies to contain the doping problem. An extensive testing program serves as one of the main drivers to counteract the doping problem (Bloodworth and McNamee, 2010). The drive to determine the predictors of doping intention has put the psychological factors that are related to doping into focus. The researchers have grappled with the conundrum in sport where the athletes still engage in the use of performance enhancing drugs (PEDs) even when they are aware that there may be health risks involved and the detrimental consequences for their sport careers if they are caught using the PEDs.

This article primary aimed to gain a deeper understanding of why an ego-orientated participant can be more susceptible to the use of PEDs or doping. Within the broad field of psychology, the meaning of the ego concept can significantly change depending on the paradigm and the specific theoretical framework as a point of departure. The notion that the ego-orientated participants are more susceptible to doping was established by Nicholls (1989) with his original work on the goal orientation theory. The task-orientated participants may be less likely to dope, because it means undermining themselves and devaluing their personal accomplishment (Ring and Kavussanu, 2017). In contrast, the approach of the ego-orientated participants of “when winning is everything it is worth doing anything to win” (Nicholls, 1989, p. 133) reveals their vulnerability and susceptibility to the use of PEDs or doping. Research within the goal orientation theory revealed mixed results. There is research that has linked to the ego-involved participants and the ego-involving motivational climate with the use of PEDs (Steyn et al., 1997; Allen et al., 2015; Mwangi et al., 2019). Furthermore, the proclivity to bend the rules and unsportsmanlike attitudes are linked to the ego-involved participants (Duda et al., 1991; Steyn et al., 1997). However, a meta-analysis by Ntoumanis et al. (2014) indicated that an ego orientation was a weak positive predictor where a task orientation was a weak negative predictor of doping intention. It is important to note that this meta-analysis primarily focused on the dispositional tendencies to be task and ego involved within the performance-evaluative context (Nicholls, 1989). According to Nicholls (1989), behavior can be regulated in any achievement context by the achievement goals that are adopted in that specific performance situation. Thus, ego and task involvement can be perceived as an expression of the dispositional ego and task orientation. Ring and Kavussanu (2017) contributed significantly to research in this area by making use of an experimental design where the achievement goals were manipulated by means of creating hypothetical scenarios with the college athletes to determine the likelihood of doping. The uniqueness of this research is because that it focused on goal involvement that highlight a situational motivation state, which can be a stronger predictor of doping intention than the more dispositional goal orientation. This provides the first research of this kind that shows the goal involvement can be a strong predictor of the likelihood of doping. The effect size in this research for doping likelihood was medium-to-large in magnitude. From the above-mentioned research into consideration, it can be concluded that the ego-involved participants which are externally regulated display a more maladaptive set of responses that increase a likelihood of doping. The reason for the positive relationship between ego orientation and the susceptibility to use PEDs can be better understood by not only conducting a conceptual analysis, but also by tracing the historical origins of the ego concept, as well as the core meanings attached to the ego concept.

To achieve this aim, a literature study was undertaken making use of the scholarly works by experts in the field that compiled their work from the original texts combined with the work of linguistic experts that conducted original translations. This approach can be viewed as a new frontier to explore the deeper meanings of the concepts by tracking the concepts back to their origins and specific context where the concepts were created. The striking similarities between the attached meanings to the ego concept as defined within the goal orientation theory (Duda and Pensgaard, 2002) and the core themes that can be connected to the ego concept as contextualized in the Sanskrit within the Hindu tradition, were identified in the well-known historical work of the Bhagavad Gita (Versfeld, 1991; Mitchell, 2000). In addition, the concept of ego is well-presented in the Buddhist tradition (Mannion and Andersen, 2015). The ego concept that is in line with the meaning of the ego within the Buddhist tradition also resurfaced in the original work of the 12th century Christian German theologian, Meister Eckhart, in his explication of the *having mode* and the *being mode* (Fromm, 1976). These conceptual similarities also resonate throughout the Taoist movement, which includes the works of Lao Tzu, Chuang Tzu, and Lieh-Tzu, the most prominent and leading figures in the Taoist movement. It must be mentioned that the ego concept was only used in the Hindu and Buddhist tradition and in the works of Meister Eckhart, while the attached meanings to the ego were well-articulated by the Taoists. The methodology of this article follows the same approach as the qualitative studies where direct verbatim quotations from the narratives are provided to substantiate the theme that emerged (Jones, 2015). In the same way, this article relies upon the direct quotations to support the argument pertaining to the conceptual meanings of the ego. The starting point of this article will be to define, explain, and analyze the ego concept as contextualized in the goal orientation theory.

The goal orientation theory is based on the creative and original works of Ames (1992) and Nicholls (1989) who worked from the assumption that the achievement situations are characterized by a certain degree of task and ego involvement, which affect the achievement goals that are adopted. According to Duda and Pensgaard (2002, p. 51), ego involvement is “principally concerned with the demonstrating superior skill to other competitors. They focus upon establishing competence and skill and, because of this objective; the activity at hand is really a means to an end, rather than an end in itself. Consequently, in contrast with an athlete who is task-involved, an ego-involved athlete is less likely to be *in the moment*.” This quote strikes the core of an ego-involved participant that focuses primarily on the outcome and the task is only a means to achieve the outcome. The ego-involved participant maintains a perception of abilities by the successful outcomes. The second external mode of measuring success and failure and maintaining perceptions of high ability for an ego-involved participant, involve social comparison (Roberts et al., 1999; Harwood, 2005). The demonstration of ability and superior skill for an ego-involved participant is to outperform the opponent with the least effort (Roberts et al., 1999). The view of an ego-involved participant of their own abilities is vulnerable, because their ability is perceived to be stable and fixed (Harwood, 2005). This fixed mindset (ego-involved) vs. the growth mindset (task-involved) can also be linked to a plethora of research that was conducted by Dweck (2000, 2005) on the self-theories, which also connect the fixed mindset with the problem of cheating. It is for this reason that an ego-involved participant is inclined to use the least effort to achieve a goal, because exerting effort can be seen as lack of ability. If an ego-involved participant cannot demonstrate ability, the maladaptive achievement behaviors may manifest. This includes the susceptibility to use the PEDs and the manifestation of other behaviors, such as unsportsmanlike behavior (Duda et al., 1991), aggression (Dunn and Dunn, 1999), bending the rules (Steyn et al., 1997), and avoiding the challenges, all of which (Roberts et al., 1999) are linked to an ego-involved participant.

In contrast, a task-involved participant is primarily focused on the task as an end in itself and the main goal is to master the task, improve the skill level of an individual, and experience personal improvement by learning and giving the best effort possible. The task-involved participant sense of high competence is rooted in self-referenced criteria and, therefore, is more intrinsically involved in the sport participation (Duda and Pensgaard, 2002). The research of Roberts and Ommundsen (2007) indicated that when the perceived mastery criteria are operative in a sport context, the participants are more engaged in the task, have higher performance satisfaction and enjoyment, better peer relationships, motivation is optimized, burnout and dropout are reduced, and cheating is lessened. In short, the task-involved participant reveals higher levels of psychological well-being.

The conceptual analysis of an ego-involved participant, which can be contrasted with the task-involved participant, can be used as a starting point to trace back the essential meanings to the original manifestations in historical works that were written ~2,500 years ago. The main goal of uncovering the derivations of the meaning of the ego concept is to deepen the understanding of why an ego-involved participant is more susceptible to using the PEDs or doping.

## Historical Roots of the Ego and Outcome Orientation

Within the applied sport psychology context, the most well-known psychological principle that is recommended for optimal performance in sport is to focus on the process (task) and not the result (outcome). According to McCluggage (1983, p. 68), “Letting go is means oriented; it is playing the process, not the result.” This is one of the most prominent psychological principles for the high-level performance in sport. Even the high-level sport participants know the detrimental effect “When winning is critical, the last thing to think about is winning” (Goldberg quoted in Potgieter, 2007, p. 73).

This core principle for effectiveness can be traced back to the writings of Chuang Tzu 2,500 years ago in his description of “the need to win” (Merton, 1997, p. 107). The following words of Chuang Tzu capture the essence of playing the process and not the result and explain the consequences when the archer becomes outcome orientated: “When an archer is shooting for nothing, he has all his skill. If he shoots for a brass buckle, he is already nervous. If he shoots for a prize of gold, he goes blind or sees two targets—he is out of his mind!” (Merton, 1997, p. 107). This core principle is not only reverberated throughout almost all the writings of Chuang Tzu but also echoed by the two other leading Taoists of that time, Lao Tzu and Lieh-Tzu. Lao Tzu captures the essence of this principle in Verse 30 of the Tao Te Ching by stating “A knower of the Truth does what is called for then stops, he uses his strength but does not force things in the same way, complete your task, seek no reward, make no claims” (Star, 2001, p. 38). Lieh-Tzu aligns himself with this central theme by stating, “When we do not anticipate success and failure, we will be prepared to accept any outcome. We will not be terribly overjoyed if things turn out the way we want, but we will not be miserable should things run amok” (Wong, 1995, p. 180). This essential principle pertaining to the process/outcome dynamics does not only have roots in the ancient wisdom of the Taoist and the Hindu tradition but also prominent in the Buddhist tradition, which is also the origin of the mindfulness movement in modern psychology (Hanh, 2008; Suzuki, 2011).

This psychological principle was not only a central theme with the Taoist and Buddhist movement, but it is even more prominent as a life and spiritual principle in one of the most literary and spiritual masterpieces of the world created in almost the same time period as the Taoist movement, namely the Bhagavad Gita, which was part of the core text of the Hindu tradition. To focus on the task (process) and not the outcome is a golden thread or theme throughout the text of the Bhagavad Gita. The statements such as “You have the right to your actions, but never to your actions' fruits. Act for the action's sake. And do not be attached to inaction. Self-possessed, resolute, act without any thought of results, open to success and failure” (Mitchell, 2000, p. 55) elucidate this core principle. Gandhi, the well-known spiritual leader of India, made a lifelong study of the Bhagavad Gita and was his main framework from which he found guidance for his spiritual approach to life. He made the following statement pertaining to the core principle of the Bhagavad Gita: “Renunciation of the fruits of action is the center around which the Gita is woven. It is the central sun around which devotion, knowledge, and the rest revolve like planets” (Gandhi appendix in Mitchell, 2000, p. 20). Gandhi also stipulated the ramifications of being outcome orientated and focused only on the results. The following words of Gandhi (Gandhi appendix in Mitchell, 2000, p. 217) explicate the essential ramifications: “He who is always brooding over results often loses nerve in the performance of his duty. He becomes impatient and then gives vent to anger and begins to do unworthy things; he jumps from action to action, never remaining faithful to any,” “everything is right in his estimation, and he therefore resorts to means fair and foul to attain his end” (Gandhi appendix in Mitchell, 2000, p. 216–217). This statement by Gandhi can be linked to almost all the maladaptive behaviors of the ego-involved participants that have been identified by the researchers (Duda et al., 1991; Roberts et al., 1999) using the goal orientation theory, namely, aggression “vent to anger,” lack of motivation, “jumps from action to action,” tendency to bend the rules, and most importantly the susceptibility to use the prohibited substances or doping “resorts to means fair and foul to attain his end” (p. 216–217).

The question that arises from this discussion pertaining to the process (action, task)/outcome (the fruits of actions and results) dynamics is so crucial in the Hindu tradition and in the Taoist movement, that is why the action for the sake of action is elevated to a kind of golden standard for living and spiritual practice. Versfeld (1968, p. 162) made an in-depth study of the ego in Indian thought. He concluded that the Hindu tradition perceived the ego, “*Ahamkara,”* as the source of suffering. The real subject or consciousness, “*Purasa,”* remains unknown. Ego desires that are the source of suffering also correlate with the Buddhist concept of ego that represents the derailed clinging and craving (Mannion and Andersen, 2015). The reason why the outcome and results (the fruits of actions) must be devalued is because the outcome feeds the derailed desires of the ego and promotes ego-aggrandizement that may reinforce the strength of the ego and human suffering. The process/outcome dynamics manifests in a fundamental shift in the relationship of an individual to either the process or the outcome. This fundamental shift from the process toward the result and the significant change in the relationship toward the world and people are effectively illustrated by the flow expert, Csikszenthmihalyi (1990). Csikszenthmihalyi (1990) indicates that a self-centered approach can radically change the relationship to the world and how an individual relates (ego) desires to the world and people. “For such a person everything is valueless in itself. A flower is not worth a second look unless it can be used; a man or a woman who cannot advance one's interests does not deserve further attention” (Csikszenthmihalyi, 1990, p. 84). This fundamental difference in relating to the world and people is also resonated in the *I-Thou* and *I-It* relationship that was developed by Buber (1970). The difference between the *I-Thou* and the *I-It* was translated to a sport setting by McCluggage (1983) in reference to the good and the poor skier in the following description: “The poor skiers (with I-It relationship) fight the mountain, attacking it with their tiny poles, their miniature spirits, and slashing at it with their edges. The good skiers join the mountain, commune with it, go with it. The good skiers have an I-Thou relationship with the mountain; there is union” (McCluggage, 1983, p. 11). According to the philosophers and the historic works of the Sages, it seems that this principle *to act for the sake of act* is deeply embedded into the core and original nature of the humans. The work of Chuang Tzu (2500BC) emphasizes this primordial principle throughout his work when he refers to the “Superior Man,” “Noble Minded Man” (p. 18), and “The Kingly Man” (p. 72) that live in accordance with this principle and all the actions bear the mark of the “right way” (Merton, 1997, p. 31). According to Meister Eckhart, this primordial principle can even be explained on a deeper meta-physical level by stating that “acts for the sake of action” can be directly related to the great Creation act itself (Suzuki, 2007, p. 3). The reason why this principle can be perceived as a blueprint for quality living and quality creation is the fact that the Creator himself “acts for his own sake” and “acts for the sake of action” (Eckhart quoted in Suzuki, 2007, p. 3).

Another argument from a different perspective encompasses of the historical works that have been used to enlighten the importance of the process/outcome dynamics in relation to the explanation of why an ego-involved participant is more susceptible to cheating and doping, is to be found in the phenomenon of play. The philosophical inquiry into the phenomenon of play revealed that the core characteristic of play is to be found in the process of the activity or rather to do the activity for the sake of doing it (Huizinga, 1988; Suits, 1988). This essential play quality can also be classified as an autotelic activity (Suits, 1988). Within the flow phenomenon, Csikszenthmihalyi (1990) emphasized the importance of autotelic activities. An autotelic activity refers to a self-contained activity with no future benefit, because the activity itself is the reward, which in a sense defines the essence of play (Csikszenthmihalyi, 1990).

The work of Piaget in extensive investigation into the play phenomenon can enlighten the relationship between play and moral decision-making (Piaget, 1932). Piaget and co-workers discovered in their investigation of the French school boys that play with marbles within the school setting and apply the rules of the game diligently with absolute care not to break the rules of the game. This strong tendency for fair play is also in accordance with the insight of Huizinga (1988) that the spoil sport, which is equal to the false player or the cheat is immediately banned from the play, because the spoil sport destroys the play-world itself. Versfeld (1968) is in line with the view of Huizinga and explains that people play not to uphold the law (rules), rather than the rules that provide a setting for more freedom for the players to express joy in play and the rules by which play flows spontaneously from the phenomenon of play itself. The question that arises from the work of Piaget (1932) and an enquiry of the philosopher of the sport phenomenon is that play and moral decision-making (abiding by the rules of play) are intertwined and that the decrease of the play quality in the human actions also coincide with the decrease of the quality of moral decision-making (deviating from the rules). A goal orientation theory indicates that the ego-involved participant approaches the activity (process) as “a means to an end, rather than an end in itself” (Duda and Pensgaard, 2002, p. 51), is it not reasonable to raise the question that lack of play can be associated with the tendency to cheat and make use of PED. Another hypothetical question that can be raised from this argument pertaining to the intertwined nature of play and moral decision-making is that a task-involved participant who is intrinsically engaged in the task itself and more in the moment, because of the joy in participation, has more play essence that assists a higher level of moral decision-making with a lower tendency to cheat and using the PEDs. These questions do not need to be answered in the context of this article but are only hypothetical notions that are raised to strengthen this argument and may be worthwhile exploring in future research.

## THE Ego, Social Comparison, and the Fixed Mindset

One of the essential features of an ego-involved participant is the perception of abilities as stable and fixed. This fixed mindset of own abilities necessitates the positive perception of abilities that must be maintained through the external norms of positive outcomes and social comparison (Harwood, 2005). The ultimate success for an ego-involved participant is to beat an opponent with the least effort. An ego-involved participant will maximize the perception of ability through winning and reduce effort that indicates high athletic ability. It is for this reason that effort can be viewed as negative because exerting effort can indicate the lack of ability (Roberts et al., 1999; Harwood, 2005).

These dynamics can be attributed to a fixed mindset (entity theory) vs. a growth mindset (incremental theory), which has been extensively researched by Dweck (2000, 2005); a fixed mindset is linked to the problem of cheating. The fixed perceptions of the abilities of an individual can be traced back to the *having mode* explicated by Fromm (1976). The core essence of the *having mode* involves the ego as the most important object of our property structured view of life. The *having mode* is not only an obsession with the objects but is rather an attitude that infiltrates all the areas of life, knowledge and learning. *Having mode* individuals resist new thoughts and ideas that can disturb the fixed perception of the world (Fromm, 1976). This includes the perception of ego individuals of the abilities of oneself, which are more rigid, unchangeable, and sterile. Anything that threatens their perception of ability can have a disturbing effect on an ego-orientated individual and may develop into the maladaptive behaviors. According to Fromm (1976), another important attribute of the ego, which is the essence of the *having mode* is the problem of greed that is a natural manifestation of the *having mode*. This greed tendency has no satiation point because of the inner boredom, loneliness, and emptiness of an ego individual. Therefore, an ego individual must have more than anyone else. This tendency leads to antagonism, competition, and fear (Fromm, 1976). The following quote by Fromm (1976, p. 114) that “… there must be competition and antagonism among individuals in the struggle for getting the most. And the strife would continue even if a state of absolute abundance could be reached; those who have less in physical health and in attractiveness, in gifts, in talents would bitterly envy those who have more” indicates the unlimited desires of an ego individual. A prerequisite to determine who has the most is to compare oneself with other individuals. Therefore, a social comparison is such a prominent essential for an ego-involved participant.

## THE Ego and Mistaken Identity

Fromm (1976, p. 111) asks a significant nexus of human identification question: “If I am what I have and if what I have is lost, who then am I?” This indicates the problem when the individuals identify themselves fully with something outside of themselves on which they depend fully and invest their total identity into something they have, such as money, prestige, being a super-star worker, or sport participant. The second question that flows from this kind of identification is what will happen to me if I lose what I have, because everything that one has can be lost in the future. This fear of losing everything and being confronted by the possibility of total loss and even facing a fatal kind of abyss and nothingness can be the motivation to go to the extreme measures to protect the identity of oneself. Applying this total loss of identity to sport participation, the question arises who the sport participant will be without sport. This catastrophic fear can create a deep fear inspired motivation that can open the gateway to the extreme maladaptive behaviors, such as cheating and susceptibility to use the PEDs.

Mannion and Andersen (2015, p. 5) confirm the over-identification problem in sport, but make use of the original thoughts in the Buddhist tradition to explain why this attachment can lead to over-identification with sport and the fitness industry in the following words: “Further complicating matters is the “me” (or ego) that becomes slave-like to this clinging and craving, but this ego is not inherently real. The ego, or felt sense of “I,” is reified, and we, subsequently, become attached to and defensive of it. Exercise settings offer many examples of this clinging, craving, and ego-defense.” This over-identification problem and the link to susceptibility toward the drugs and alcohol are identified by Brewer and Petitpas (2017) and Hale and Waalkes (1994), as well as the problem of identity foreclosure among the young adolescent participants (Marcia, 1966, 1993; Nkonki, 2021).

In the elicitation of the *having mode* by Fromm (1976), the ego as a bundle of desires is the core driving force in the manifestation of the *having mode*. This original and significant link between the ego and the *having mode* has been elucidated by Meister Eckhart in the 12th century (Fromm, 1976). The concepts of ego of Meister Eckhart, which are identified as “Eigenschaft as Ich-bindung or Ich-sucht as egoboundness” (Eckhart quoted in Fromm, 1976, p. 68–69) are concerned with the same desires that are also prominent in the Buddhist tradition that equals attachment and craving. This strong resemblance of Meister Eckhart's analysis of the ego as a concept and its manifestation in the *having mode* with the classic traditional views of the ego are also confirmed by Daisetsu Suzuki, a Japanese scholar, who is well-known for knowledge of the Eastern, Hindu, and Buddhist traditions, as well as the work of Meister Eckhart (Suzuki, 2007). A striking feature of the ego phenomenon is that it is coherently defined and described with the same essential features not only by Meister Eckhart and Fromm, who expounded on the ideas of Meister Eckhart, but is also articulated in the Buddhist and Hindu traditions (Versfeld, 1991).

A surprising discovery was that these coherent perceptions of all the metaphysical traditions also correspond significantly with the *content self* (conceptualized self) and the *context self* (transcended self) that are two of the three selves (*context self*, *process self*, and the *content self*) important components of the self as formulated in the Acceptance Commitment Therapy (ACT) (Bach and Moran, 2008). Interestingly, this *content self* is also closely linked to the problem of attachment and psychological inflexibility. This tendency is understandable because the definition and description of the *content self* accurately corresponds with the definition and descriptions of ego in the Buddhist and Hindu traditions, as well as the ego defined within the *having mode* (Fromm, 1976). Within the Christian tradition, the closest concept to the ego that corresponds with the Buddhist and Hindu traditions is the concept of “pride, which is the idolatry of the self” (Versfeld, 1991, p. 160). St Augustine, the Christian church father who was known for deep understanding of the human desires, captures the essence of the egoistic self-love with the Latin concept of *amor concupisentiae* (Versfeld, 1991, p. 223). In contrast, the *context self*, which is essential in the therapeutic process and especially for the clients to improve their willingness for acceptance, is a prerequisite for the effectiveness of the ACT approach. The *context self* or the “self as perspective is transcended in that it has no form or verbal content” (Bach and Moran, 2008, p. 10). This formlessness with no content nature of the *context self* as the concept or rather non-concept resonates with the nothingness concept that can be traced throughout the traditions that have been referred in this article. The Buddhists refer to the Void and the nothingness as “nameless nothingness” (Suzuki, 2007, p. 44) that encompasses everything. Meister Eckhart makes use of words, such as “desert,” “stillness,” “silence,” and “nothingness” to explain the nature of the *being mode* (Suzuki, 2007, p. 10), while the Taoists, such as Lao Tzu, refer to “pure nothingness” as the “Way” that is characterized by emptiness “… that yet may be drawn from without ever needing to be filled. It is bottomless” (Suzuki, 2007, p. 14). Within the Hindu tradition, “the *Purusa* (the Great Self), the real subject or consciousness, remains unknown” (Versfeld, 1991, p. 162). All these references from the spiritual traditions coincide with the formlessness with no content of the *context self*. The ACT is one of the leading developments in the mindful and acceptance approach and that is part of the third-wave cognitive development which is supported by a plethora of research. These articles provide substantial evidence of the effectiveness of this approach. The possibility to trace back the *concept self* and the *context self* as the essential components of an effective psychological approach to their historical roots is another indication that the psychological phenomena in modern psychology can be strongly linked to the historical writings that were created in the spiritual realm of human existence.

## Conclusion

This article primary aimed to elicit a deeper understanding of the psychological motives of athletes that have a proclivity toward cheating and doping. The concept and meanings attached to an ego-involved participant as formulated in the well-established goal orientation theory reveal striking similarities with the ego as elucidated in the historical metaphysical and spiritual writings, 2,500 years ago. According to Suzuki (2007), a modern psychology has done away with the ego entity and opted for a workable hypothesis to refer to the functionality and the manifestations of the ego and not to the ego as a separate entity. An analysis of the concept of the ego as an entity itself was articulated in the major metaphysical and spiritual practices of the Hinduism, the Buddhism, and the original concept of the *having mode* as a manifestation of the ego desires elucidated by Meister Eckhart and expounded by the creative interpretations of Fromm (1976). This analysis of the ego within the major spiritual traditions created a fusion zone between the modern psychological theories, approaches, concepts, and spiritual traditions of the past. The fusion zones that psychology developed with physiology (psycho-physical) and sociology (psycho-social) are well-established and recognized as the interdisciplinary relations. The psycho-spiritual fusion zone is not that well-acknowledged and developed, because of the metaphysical nature of the spiritual realms. This psycho-spiritual interchange can be a frontier that can enrich the field of psychology, as well as the field of theology. Hayes (1984), a leading proponent in the ACT approach, in his article “Making sense of spirituality” provides a well-substantiated argument to conduct a behavior analysis of the distinction between the matter and spirit from a behavior theory viewpoint. Hayes believes that the behavior analysis of spirituality can enrich several topics in the psychology, such as self-awareness and therapeutic approaches. This may be a relatively new frontier by revisiting and exploring the spiritual traditions that existed for millennia. This endeavor can only enrich the existing field of psychology.

## Author Contributions

BS conceived idea. BS and KN were involved in writing the article. All authors contributed to the article and approved the submitted version.

## Conflict of Interest

The authors declare that the research was conducted in the absence of any commercial or financial relationships that could be construed as a potential conflict of interest.

## Publisher's Note

All claims expressed in this article are solely those of the authors and do not necessarily represent those of their affiliated organizations, or those of the publisher, the editors and the reviewers. Any product that may be evaluated in this article, or claim that may be made by its manufacturer, is not guaranteed or endorsed by the publisher.
